# Pseudotext Injection and Advance Filtering of Low-Resource Corpus for Neural Machine Translation

**DOI:** 10.1155/2021/6682385

**Published:** 2021-04-11

**Authors:** Michael Adjeisah, Guohua Liu, Douglas Omwenga Nyabuga, Richard Nuetey Nortey, Jinling Song

**Affiliations:** ^1^School of Computer Science and Technology, Donghua University, Shanghai, China; ^2^School of Information Science and Technology, Donghua University, Shanghai, China; ^3^School of Mathematics and Information Technology, Hebei Normal University of Science & Technology, Qinhuangdao, Hebei, China

## Abstract

Scaling natural language processing (NLP) to low-resourced languages to improve machine translation (MT) performance remains enigmatic. This research contributes to the domain on a low-resource English-Twi translation based on filtered synthetic-parallel corpora. It is often perplexing to learn and understand what a good-quality corpus looks like in low-resource conditions, mainly where the target corpus is the only sample text of the parallel language. To improve the MT performance in such low-resource language pairs, we propose to expand the training data by injecting synthetic-parallel corpus obtained by translating a monolingual corpus from the target language based on bootstrapping with different parameter settings. Furthermore, we performed unsupervised measurements on each sentence pair engaging squared Mahalanobis distances, a filtering technique that predicts sentence parallelism. Additionally, we extensively use three different sentence-level similarity metrics after round-trip translation. Experimental results on a diverse amount of available parallel corpus demonstrate that injecting pseudoparallel corpus and extensive filtering with sentence-level similarity metrics significantly improves the original out-of-the-box MT systems for low-resource language pairs. Compared with existing improvements on the same original framework under the same structure, our approach exhibits tremendous developments in BLEU and TER scores.

## 1. Introduction

Access to large-scale parallel corpora plays a substantial role in training a high-quality statistical and neural MT (SMT & NMT) systems. However, such corpora are not freely available for many language translation pairs. Constructing a high-quality parallel corpus is time-consuming, which requires financial resources and professional translation of a considerable quantity of text. While large monolingual corpora are readily available, numerous of the already available large-scale parallel corpora are limited to specific language domains. In contrast to major Western languages, most African languages are very low-resourced [1].

A quality pseudoparallel sentence is predominantly significant as a low-quality parallel corpus will reduce NMT's performance compared to SMT [2]. However, numerous methods have been used to create pseudoparallel sentences from a monolingual target corpus with percentage points of accuracy improvement. Many work lines are still much underexplored. Some of these approaches include using a monolingual corpus of the source language and its automatic translation to generate pseudoparallel sentences [3]. Sennrich et al. [4] engaged synthetic-source sentences and achieved a significant accuracy via automatic translation of a monolingual corpus of the target language into the source language. They thereby concatenate the acquired pseudoparallel text to expand the training corpus. The approach successfully understands the conditional language model from the monolingual corpus of the target language. The result of their work is encouraging; however, they did not entirely exploit the eminence of the pseudoparallel corpus as they only investigated comparatively large-scale parallel corpora language pairs.

In this work, we propose to inject pseudoparallel corpus to the training data via iteratively applying the neural Transformer for back-translation. We carefully studied sentence-alignment metrics for filtering before and after successful round-trip translation. The assumption is that if the target sentence and its round-trip translation are parallel, then, presumably, the synthetic source sentence fits the monolingual target sentence. Hence, we can incorporate it into the filtered synthetic-parallel corpus. The additional synthetic sentences augmented the training data, thereby projecting it to what we call medium-resource language pair by iteratively applying the neural Transformer and advanced filtering techniques for round-trip translation. It is often perplexing to learn and understand what a high-quality corpus looks like in low-resource conditions, mainly where the target corpus is the only sample text of the parallel language. Therefore, we performed unsupervised measurements on each sentence pair, engaging squared Mahalanobis distances, a filtering technique that predicts sentence parallelism [5]. In low-resource language pairs, in which only low-accurate MT systems can be used, the translation quality degrades when a synthetic-parallel corpus is naively used. For instance, as a result of back-translation and round-trip translation, the synthetic target data is populated with sentence duplication, near-duplication, and sentence agreement equivalent to chance as well as noise sentences.

Listed below are the main contributions of our research:

1. We implemented an iterative neural Transformer with different parameter settings to expand the training data. The data are expanded by injecting pseudoparallel corpus obtained after translating a monolingual corpus from the target language.
2. We show that predicting sentence parallelism on the target monolingual and the source-synthetic data, and handling duplication, and sentences agreement equivalent to chance before round-trip translation, serves as a supervisory signal to learn what a “clean” corpus looks like.
3. Additional sentence-level similarity metrics like Cohen's Kappa, average alignment similarity (AAS), and maximum alignment similarity (MAS) after round-trip translation are worthwhile for low-resource language pairs.
4. We observed that the combined methodologies successfully obtain a high-quality pseudoparallel corpus for low-resource language pairs with percentage points of improvement in accuracy.
5. The synthetic-parallel corpora (https://github.com/Madjeisah/tw-parallel-lg-corpus) are publicly released for noncommercial use.

To establish our approach's effectiveness, we experimented on multiple language pairs with a diverse amount of accessible parallel corpus. English⟷Twi (a new language pair), English⟶Afrikaans, Xitsonga and Setswana, and Japanese⟶Russian are low-resource language pairs and German⟶English is a high-resource language pair. Although the baseline approach by many authors [4, 6] is sufficient for high-resource language pairs, considering the number of parameters, NMT systems tend to overfit on small training data. Hence, the best practice is to expand the training data using the filtered synthetic-parallel corpus, especially in low-resource language situations where the target corpus is the only sample text of the parallel language.

We organize the rest of the paper as follows: Section 2 discusses the related work to improving low-resource situations. We give detailed steps of the system architecture in Section 3. Experimental results on the proposed model and discussions are presented in Section 4, and, finally, Section 5 outlines the conclusion and future directions.

## 2. Related Work

Many approaches [3, 6–10] use monolingual data to boost translation quality to fix data sparsity in MT; specifically, by training a translation model from a generated pseudoparallel corpus formed from a monolingual in-domain corpus, Bertoldi and Federico [9] addressed the domain adaptation issue. Hsieh et al. [10], in their early work, developed a pseudoparallel corpus for cross-domain adaptation based on patterns learned from the target source and monolingual target in-domain corpora. They carried out the filtration of “relatively more precise” translated phrases manually and employed them to refine the language processing model. Other multiple techniques use iterative methods to optimize NMT exploiting pseudoparallel corpora [11, 12]. Zhang et al. [6] iteratively implemented both source and monolingual target corpora to strengthen an NMT system. They generated synthetic sources by sampling and adding noise to beam outputs [13], which further enhanced high-resource NMT. For every individual target sentence in the corpus, Imamura et al. [14] sampled several sources to boost the encoder and attention process, which led to an improved quality translation. Experiments on reasonably high-resource language pairs were, however, performed as well. Niu et al. [15] engaged in continuous training of an augmented high-resource parallel corpus, which finally enhanced a bidirectional NMT model. Likewise, we inject synthetic-parallel corpus in this analysis by translating a monolingual corpus from the target language based on bootstrapping with different parameter settings. Automatic filtering, in comparison, is also introduced to the pseudoparallel corpus produced. We carried out experiments on both low- and high-resource lexical items to emphasize the reliability of the preceding filtered synthetic-parallel corpora generated from the target language.

In domain adaptation [16, 17] for phrase-based SMT schemes, data filtering is frequently implemented. Imankulova et al. [18] derived sentences from enormous corpora to refine the language processing method and the translation [18–20]. To learn what a “clean” corpus looks like, Littell et al. [5] measured conceptually comparable to pointwise mutual information inherent of sentence pairs in terms of Mahalanobis distances rather than actual probabilities. The approach cogitates, respectively, the sentence pair as a draw from the distribution of high-dimensional vectors where a “strange” sentence pair is one whose draw was unlikely compared to the probability of depicting independent sentences module. The studies by Imankulova et al. [18] and Yildiz et al. [20] are closely related; Yildiz et al. [20] in their proposed model constructed a quality estimator using a bilingual dictionary to achieve higher and quality parallel sentence pairs. With a small, high-quality corpus, they obtained an enhanced translation efficiency and decreased time complexity. The filtering data strategy is accomplished via the mentioned method, thus measuring the similarity between the source and target sentences. Imankulova et al. [18] achieved a successful measurement of similarity between monolingual and synthetic target sentences without any external dictionaries. They successfully improved low-resource Russian⟷Japanese language pair by expanding the training data using filtering the pseudoparallel corpus and employing quality estimation based on sentence-level round-trip translation. The authors Van der Wees et al. [21], through dynamic data selection in the training of an NMT framework, utilized language processing techniques from the source and target sides of in-domain and out-of-domain data to determine cross-entropy scores. Hereafter, the training information is efficiently sorted and filtered. However, the present analysis also indicates that the authors adopted round-trip translation to aid the filtering of data, taking its context into account.

Besides these, a dual-learning technique proposed by He et al. [22] concurrently trains two models via a reinforcement learning mechanism. The method engaged the monolingual information of both source and target languages and produced detailed feedback signals to train the translation involved techniques. By increasing their efficiency, the dual-learning approach has shown a promising result in mitigating noisy data. However, in our method, we try to exclude noisy duplication and near-duplication data. Furthermore, they believed that a high-recourse language pair would “warm-start” the reinforcement learning process. Simultaneously, we aimed at low-resource language pairs in our study, where it is not easy to achieve perfect high-quality seed of NMT models. In a zero-shot situation, the unsupervised strategy by Artetxe et al. [23] and Lample et al. [24] was beneficial by leveraging only monolingual corpora and back-translation. Nevertheless, we concentrate on optimizing the effectiveness of the current small parallel corpora opening the potential study of the application of present unsupervised MT methods.

The study by Imankulova et al. [18] is similar to our study in terms of iterative bootstrapping; however, they are different in implementation. Duplicate and near-duplicate sentences were avoided by measuring the inter-annotator agreement on the correctness of the target monolingual and the synthetic source data using Cohen's Kappa statistics. We furthermore performed unsupervised measurements on each sentence pair engaging the squared Mahalanobis distances by Littell et al. [5] to predict sentence parallelism before round-trip translation. We also computed the similarity between the monolingual target and synthetic target sentences. Compared with existing improvements on the same original framework under the same structure, our approach exhibits tremendous developments in BLEU and TER scores with state-of-the-art performance.

## 3. System Architecture

Previously, we detailed the modeling of massively parallel Bible corpus based on Twi, a common Ghanaian language, to a handful of languages [25]. The work discussed the common issues encountered in obtaining, processing, converting, and formatting the corpus and the latent desire for NLP success. We stored the sentence-aligned data in various files based on Twi to the selected language pairs with a tab-delimited separation, where verses with the same line number in a line pair are mappings of each other. While that was dedicated to corpus development, this work gears towards how NLP can be scaled to low-resourced languages to improve MT performance.

### 3.1. Back-Translation

**Figure 1:**
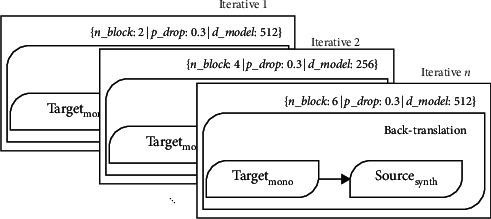
An iterative neural Transformer for extra-pseudo-parallel corpus.

Our system shares the same skeleton system architecture as the one presented by Imankulova et al. [18] and Littell et al. [5] but differs in the execution. As shown in Figure 1, the first module is to iterate the neural Transformer for back-translation to expand the training data. We varied different layers and models of the Transformer for various iteration processes to reduce training variance and selected the best five scores. See Table 1. We used the hyperparameters described by Vaswani et al. [26], except for varied batch size and numbers of layers and models. We adopted a constant dropout of 0.3 combined with a weight constraint of 0.5 to combat overfitting for iterative back-translation. Then, we predicted sentence parallelism on the target monolingual and the synthetic source data, where we performed some preprocessing and postprocessing to avoid duplicate and near-duplicate sentences. Thus, to avoid duplicate and near-duplicate sentences, we measured the inter-annotator agreement on the developed corpus's correctness of the target monolingual and the synthetic source data using Cohen's Kappa. The interpretation of Cohen's Kappa [27] on the strength of agreement states that when your data raters' precision is 0.81–0.99, it is a near-perfect agreement, and when it is 1.00, it is a perfect agreement. Hence, we consider a precision score within this range as duplicate or near-duplicate. However, not all near-duplicate precision scores were avoided as we carefully performed a human evaluation on such data. Also, sentences agreement equivalent to chance was avoided. See Table 2.

**Figure 2:**
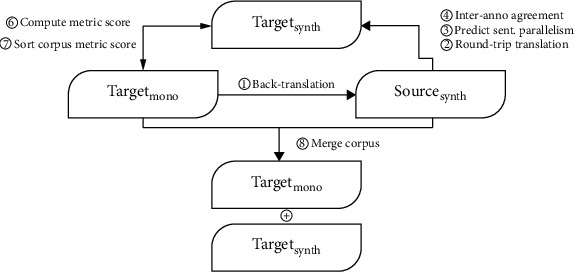
The entire architecture for creating and filtering the synthetic-parallel corpus via round-trip translation.

Each back-translation iteration involves the following steps (Figure 2):

1. “Model training”: train source-target pseudoparallel corpora with an iterative Transformer with varied parameter settings to obtain the source-synthetic data.
2. “Model selection”: run an evaluation at every epoch during the training process by hypothesizing translation and calculating the BLEU score to select the best model on the development set based on the recorded BLEU score on the source-synthetic data. If there is no improvement over the preceding iteration, then terminate the process and return to the different parameter settings.
3. “Prediction”: predict sentence parallelism on the monolingual target and the source-synthetic data.
4. “Handle duplication”: measure the interannotator agreement on the corpus's correctness of the monolingual target and the source-synthetic data.
5. Repeat steps 1 to 4.

The second module is a round-trip translation to obtain the synthetic target data. We proceed by computing the metric score to check the similarity between the monolingual and the synthetic targets. Notably, we use the AAS and MAS proposed by Song and Roth [28].

Below are the steps of the second module of the proposed method:

1. Use a round-trip translation of the synthetic source sentences in a source-target direction to acquire a synthetic target sentence
2. Compute the sentence-level similarity metric scores between the monolingual target sentences (reference) and the synthetic target sentences (candidates)
3. Filter out sentences with low scores by sorting the reference and candidates sentences in a descending order
4. Immerge the filtered synthetic target sentences and the monolingual target sentences to expand the training data, hence the term pseudotext injection

The entire architecture for developing the synthetic-parallel corpus via round-trip translation is illustrated in Figure 2.

### 3.2. Metrics for Sentence-Level Similarity Check

Four extensive similarity metrics were used for sentence-level filtering, namely, the squared Mahalanobis distances, Cohen's Kappa statistics, AAS, and MAS. The squared Mahalanobis distances metric has shown robustness in predicting sentence parallelism on the monolingual target and the source-synthetic data. These metrics require back-translation.

Cohens' Kappa was engaged to avoid duplicate and near-duplicate sentences. Unlike the squared Mahalanobis distances, the AAS and MAS metrics require round-trip translation to compute the similarity of the monolingual target and synthetic target based on distributed representations of the sentences.

Let us denote *y*=(*y*_1_,…, *y*_*i*_) as word vectors for a monolingual target sentence and *y*′=(*y*_1_′,…, *y*_*j*_′) as word vectors for a synthetic target sentence. Cohen's Kappa agreement between the monolingual target *y* and the synthetic target *y*′ raters where each classifies *N* items into *C* mutually exclusive categories can be defined as

(1) $$\begin{array}{c} k\left(y,y^{\prime }\right)\equiv \frac{p\left(y\right)p\left(y^{\prime }\right)}{1-p\left(y^{\prime }\right)}=1-\frac{1p\left(y\right)}{1-p\left(y^{\prime }\right)}, \end{array}$$

where *p*(*y*) is the observed agreement of the monolingual target and *p*(*y*′) is the hypothetical probability of the chance agreement on the synthetic target. If there is a complete agreement, then *k*=1; else *k*=0, if there is no agreement other than what would be expected by chance, as given by *p*(*y*′).

The average similarity, *A*_sim_(*y*, *y*′), between the monolingual target *y* and the synthetic target *y*′ is calculated by averaging the similarities between vectors of all words taken from the two sentences pairs as

(2) $$\begin{array}{c} A_{\text{sim}}\left(y,y^{\prime }\right)=\overset{\left|v\right|}{\underset{\begin{array}{c} i=1\\ j=1 \end{array}}{\sum }}\frac{y_{i}y_{j}^{\prime }\emptyset \left(y_{i},y_{j}^{\prime }\right)}{\left\|y\right\|\left\|y^{\prime }\right\|}. \end{array}$$

Then, the maximum similarity score *M*_sim_(*y*, *y*′) computes the similarity between the most similar words *y*′ to the target *y* word as follows:

(3) $$\begin{array}{c} M_{\text{sim}}\left(y,y^{\prime }\right)=\sum_{i=1}^{\left|v\right|}\frac{y_{i}\max_{j}y_{j}^{\prime }\emptyset \left(y_{i},y_{j}^{\prime }\right)}{\left\|y\right\|\left\|y^{\prime }\right\|}. \end{array}$$

## 4. Experimental Results

We evaluated the English⟷Twi pair for MT translation task in both directions and experiments on multiple language pairs with a diverse amount of an accessible parallel corpus for our approach's effectiveness. BLEU and TER scores were recorded based on the neural Transformer and OpenNMT systems. Furthermore, we compare the translation accuracy with existing improvements on the same original framework under the same structure.

### 4.1. Machine Translation Toolkits

For the MT process, we conducted experiments using the neural Transformer (https://github.com/tensorflow/tensor2tensor) and OpenNMT (http://opennmt.net/OpenNMT/) toolkit [29] to embark on translation. We experimented on English⟷Twi, Russian⟶Japanese, English⟶Afrikaans, Xitsonga, and Setswana as low-resource language pairs and English⟶German as a high-resource language pair. We used a vocabulary size of 75 k for all the experiments and MeCab 0.996 (http://taku910.github.io/mecab) for word segmentation for the Japanese sentences. The widely used Moses (https://github.com/moses-smt/mosesDecoder) toolkit was engaged in tokenizing and truecasing for all English, German, Afrikaans, Xitsonga, Setswana, and Russian sentences. All duplicate and near-duplicate sentences and sentences beyond 80 words were excluded. The rest of the toolkits were applied in their original state.

The sentence-wise evaluation was computed using the mteval-sentence (https://github.com/odashi/mteval) toolkit, while the Gensim library was used in training the Word2vec model for estimating the AAS and MAS metrics. In building a 5-gram language model, we used the Ken Language Model (KenLM) toolkit (https://kheafield.com/code/kenlm/) for low-resource data and Statistical Language Modeling (SLM) toolkit (http://www.speech.cs.cmu.edu/SLM/toolkit.html) for a large amount of training data. Before extracting the scores, normalization feature-scaling preprocessing of MinMaxScaler was used to transform the filtering metric scores between [0, 1] for min and max range.

### 4.2. Dataset

The English⟷Twi parallel Bible corpus benchmark is a corpus retrieved from the YouVersion Bible website. The Twi corpora contain two versions, while the English ones consist of 4 different versions (i.e., the King James Version (KJV), Good News Bible (GNB), Easy to Read Version (ERV), and New International Version (NIV)). Additional low-resource monolingual Twi and Russian⟶Japanese (http://opus.lingfil.uu.se/Tatoeba.php) data were downloaded from OPUS (http://zeljkoagic.github.io/jw300/). For English-Twi, English contains 124,400 sentences, and Twi is made up of 62,200 sentences. We iterated the neural Transformer for round-trip translation with human evaluation for extra parallel-aligned data, hence storing 124,400 parallel-aligned sentences in a tab-delimited separation. Verses with the same line number in a line pair form mappings of each other [25]. For the Russian⟶Japanese and German⟶English experiments, we engaged similar corpus statistics as those of Imankulova et al. [18] and similar corpus statistics as those of Martinus et al. [30] for English⟶Afrikaans, Xitsonga, and Setswana as presented in Table 3.

#### 4.2.1. Data Preprocessing

We used the Natural Language Toolkit (NLTK) (https://www.nltk.org/), a useful tool for loading and cleaning text. It is essential to get data ready for working with machine learning and deep learning algorithms. First, we split the text into sentences and each sentence into words after the dataset's successful loading. We refined vocabularies by removing words that were used less than five times in the dataset and replaced them with an unknown token (<UNK>). In the next preprocessing step, we converted the corpus into lowercase. Finally, we tokenized the datasets with the tokenizer function and followed it by filtering out all standalone punctuation tokens for both languages.

#### 4.2.2. Back-Translation

This part reports the experiments based on an iterative neural Transformer for back-translation to expand the training data. Back-translation has been proven to be useful for achieving significant accuracy via automatic translation [4] of a monolingual corpus of the target language into the source language in both low- and large-scale parallel corpora language pairs. While the approach performs best on a high-scale dataset, it remains a mystery for low-resource language pairs. Therefore, we varied the transformer's layers and models for various iteration processes to reduce training variance and finally selected the best five score models, as shown in Table 1, for round-trip translation.

#### 4.2.3. Training Word2vec Models

Word Embedding [21] is the backbone for the efficiently performing NLP models. The algorithms require the input features as a fixed-length feature vector. Hence, Word2vec [20] maps a text or words to real-value fixed-size vectors or converts text into semantic vectors [25]. Additional data were downloaded to train Word2vec models for the English⟷Twi experiments on which we used the corpus of Agić and Vulić [31], as it is the only domain in the Twi language. Subsequently, tokenizing and eliminating sentences of more than 100 words ended up with 218,562 monolingual Twi sentences engaged in training the Twi Word2vec. For unbiased evaluation, monolingual English sentences were cleaned and sampled to match the Twi monolingual data. Similarly, we engaged the same Japanese⟶Russian and German⟶English sentences used by Imankulova et al. [18]. Additional data were downloaded from SADiLaR (https://hdl.handle.net/20.500.12185/506) to train the Word2vec model for English⟶Afrikaans, Xitsonga, and Setswana sentences.

#### 4.2.4. Training Language Models

Like the trained Word2vec model, the same English⟷Twi, Japanese⟶Russian, and German⟶English sentences were used to train the language model experiment.

### 4.3. Training

First, we used 124,400 parallel-aligned sentences for the English-Twi corpus in our experiment to engage the models. We appended extra tokens like an unknown token (<UNK>), a padding token (<PAD>), and start-of-sentence and end-of-sentence tokens (<SOS>/<\EOS>) to the outputs. We first reshuffled the corpus and used 85% as training, 2.5% validation, and 2.5% for testing. See Table 3.

The model was optimized and tuned on the training data to evaluate the models' robustness on the validation sets. For the Transformer experiment, all the hyperparameters described by Vaswani et al. [26] were used except for the batch size of 64, 4 layers for the encoder-decoder, and a dropout of 0.5 combined with a weight constraint of 0.5. OpenNMT uses a BiLSTM for the encoder-decoder with a batch of 32 and the Adadelta optimization for low-resource language pairs; the German⟶English experiment was conducted with the default OpenNMT without back-translation. To prevent overfitting and improve the model's ability to generalize, we engaged Nitish et al. [32] standard dropout rate. Weight constraints such as the max norm constraint and regularization help to avoid overfitting and eventually increase the translation accuracy on all models.

### 4.4. Evaluate Neural Translation Model

This section presents the results on the English-Twi parallel Bible corpus. Results on a diverse amount of available parallel language are described: Japanese⟷Russian, English⟶Afrikaans, English⟶Xitsonga, English⟶Setswana, and German⟶English for model comparison with existing improvements on the same original framework under the same structure. We engaged two metrics to evaluate the translation qualities of the MT systems automatically: the Bilingual Evaluation Understudy (BLEU) score [33], Translation Error Rate (TER) [34]. Our model showed excellent performance on the training dataset and was idealized to perform well on the test set. BLEU is the most common algorithm used to evaluate the quality of MT systems automatically. It computes the translated precision by counting the number of matches between *n*-grams of a machine-translated sentence and corresponding reference. The TER, a method used by MT specialists to define the amount of postediting required for translation job work, is similar to BLEU. To compute the TER, we further processed the source and reference corpus on the test set using tercom.jar (http://www.cs.umd.edu/∼snover/tercom/) as it is much faster.

Ideally, we used a separate validation dataset of 1000 to help with model selection during training instead of the test set. We processed the corpus by excluding sentences that were longer than 80 words in training. We set the vocabulary size of both source and target languages to be 50K for English⟷Twi, which covers 85%/90% of the training data source/target side. The results are recorded in both BLEU and TER, as shown in Table 4.

Table 5 outlines a qualitative comparison with existing improvements on the same original framework under the same structure performed to understand the full effects of our model and how the experiment adds new knowledge to the state-of-the-art solutions. Our approach exhibits tremendous developments in BLEU and TER score with state-of-the-art performance. The bold scores indicate the highest BLEU scores for each languages pair.

Observation from the translation accuracy suggests that the approach generated a synthetic-parallel corpus with better quality and significant improvements in low-resource language pair scenarios. Although our model could not outperform that of Sennrich et al. [4] baseline on the development and test sets, regardless of filtering metrics, it is, however, the closest in BLEU score performance as compared to other existing improvements. Also their experiment results demonstrate that the approach is useful for high-resource language pairs; however, additional filtered synthetic-parallel corpus for expanding the training data is more effective in low-resource language pairs.

### 4.5. Discussion

The results prove that our proposed method improved the translation performance in all experiments carried out on low-resource language pairs. The far-reaching engagement of different filtering metrics in various stages to create and filter the synthetic-parallel corpus clarifies that filtering notably impacts low-resource language pairs. We observe that the efficiency across various filtering metrics is consistent with trivial exceptions.

Our model architecture suggests that the predicted sentence parallelism on the monolingual target and the source-synthetic data can handle duplication by measuring the inter-annotator agreement on the corpus's correctness of the monolingual target and the source-synthetic data. During Cohen's Kappa experiment, we considered a precision score within the ticked (✓) ranges in Table 2. We carefully studied sentences marked as (✗/✓) to decide whether to add or exclude them.

In the synthetic corpus, we include neither the “agreement equivalent to chance,” “slight agreement,” nor “perfect agreement.” One may also consider the “slight agreement” interpretation. However, such precision range requires a thorough human postediting for the pseudosentences. Also, a postediting of this agreement requires another check for duplication. Such computation would somewhat reduce the speed efficiency of the model. A little work was performed on the “fair agreement” and “near-perfect agreement” as some sentences are considered fit to be engaged as synthetic corpus.  Table 6 lists examples of selected bootstrap iteration precision of the various Cohen's Kappa agreement.

We can see clearly from Table 6 that the synthetic Twi sentence from Models 1 and 2 is an incorrect translation and thus is considered as noise. When present in the corpus and used as additional training data, such noisy sentences lead to a decreased translation quality of the NMT model, especially in low-resource scenarios; hence they need removal. However, Models 4 and 5 are strongly considered, as their translation output was sufficient to be added to the training data. Model 6 was manually inspected and about 60% of the sentences were avoided. To understand what a “clean” corpus looks like in low-resource situations, like the Twi monolingual sentence, we performed unsupervised measurements on each sentence pair with the squared Mahalanobis distances that predicted parallelism on the dataset.

The experimental results show that an iterative neural Transformer with different parameter settings to expand the training data greatly improved NMT in terms of the BLEU and TER scores. Nevertheless, sentence parallelism prediction to understand each sentence pair to select the best bootstrap iteration model for a high-quality pseudoparallel corpus is highly recommended as low-resource NMT systems depend not only on the amount of training data but also on the quality. Sennrich et al. [4] presented a pseudoparallel corpus as extra data to significantly improve the NMT baseline model's performance. However, the experiment results listed in Table 5 show that the proposed filtering could not outperform their model for a high-resource language pair. Therefore, it is not effective when compared to a low-resource language scenario.

## 5. Conclusions and Future Work

Most African languages are very low-resourced. Hence, in this work, we engaged a novel iterative of the neural Transformer to expand the training data for low-resource language pairs. Further, advanced corpus filtering approaches were proposed to perform round-trip translation of a target monolingual corpus. Observation from the translation accuracy attests that our approach successfully obtains a high-quality synthetic-parallel corpus for low-resource language pairs with high percentage improvement. Therefore, suggesting that translation accuracy is determined by the size and quality of the corpus, mainly where the target corpus is the only sample text of the parallel language, experimental results on a diverse amount of available parallel corpus demonstrate that injecting pseudoparallel corpus and extensive use of different filtering metrics significantly improve the original out-of-the-box MT systems for low-resource language pairs. In a qualitative comparison with existing improvements on the same original framework under the same structure, our approach exhibits tremendous developments in BLEU and TER scores. This work's findings present the possibility of conveniently adducing one of the most important directions for the future of MT in these language pairs.

An obvious next step is to increase the dataset used to fit the model by performing human translation on daily used phrases or creating a system of large English⟷Twi sentence-alignment corpus for translation. Furthermore, inquiries are essential to evaluate the boundaries of our proposed technique. Additionally, in this work, a single Ghanaian language (English⟷Twi) pair and five different language pairs were considered, in which each contained a different amount of available parallel data with diverse domains. We plan to provide benchmark BLEU and TER scores for translation tasks between English and the remaining official Ghanaian languages to extend this research. Another domain to reflect on is evaluating our proposed technique for morphologically rich languages like Chinese, Japanese, Korean, and Slavic with the Twi language. Twi is an analytic language, and both have no clear word boundaries. The high rate of homographs in these languages causes word ambiguities, which creates queries in NMT.

## Figures and Tables

**Table 1:** Model selection for the best 5 models on the development set.

Parameter settings			BLEU score
*n*_block	*p*_drop	*d*_model	
4	0.3	512	12.11
6		512	12.04
8		512	11.59
6		256	11.62
8		256	11.38

The *n*_blocks are the number of layers engaged by the Transformer.

**Table 2:** Precision of Cohen's Kappa interpretation for the strength of agreement.

Precision score	Agreement	Acceptance
0	Agreement equivalent to chance	✗
0.1–0.20	Slight agreement	✗
0.21–0.40	Fair agreement	✗/✓
0.41–0.60	Moderate agreement	✓
0.61–0.80	Substantial agreement	✓
0.81–0.99	Near-perfect agreement	✗/✓
1	Perfect agreement	✗

**Table 3:** Statistics of English-Twi corpus.

Corpus	English↔Twi	Russian↔Japanese	German→English
Train	122,400	18,642	4,535,522
Dev	1,000	800	3,000
Test	1,000	800	3,003
Monolingual target	186,537↔91,642	75,402↔165,742	4,208,439

**Table 4:** Official translation results on English↔Twi sentence pairs.

		BLEU	TER
En-Twi	OpenNMT	19.63	50.31
	Transformer	18.36	54.51

Twi-En	OpenNMT	19.48	50.68
	Transformer	18.13	54.76

**Table 5:** Comparison with existing improvements on the same original framework.

		Metric	
		BLEU	TER
Japanese→Russian	Imankulova et al. [18]	15.56	—
	Proposed system	**18.45**	**62.13**

English→Afrikaans	Nekoto et al. [35]	19.56	—
	Martinus and Abbott [1]	20.60	—
	Proposed system	**24.09**	**58.93**

English→Xitsonga	Nekoto et al. [35]	13.54	—
	Martinus and Abbott [1]	17.98	—
	Proposed system	**19.76**	**61.22**

English→Setswana	Nekoto et al. [35]	19.66	—
	Martinus and Abbott [1]	15.60	—
	Proposed system	**21.01**	**59.96**

German→English	Sennrich et al. [4]	**29.5**	—
	Lample et al. [36]	25.2	—
	Imankulova et al. [18]	26.23	—
	Artetxe et al. [37]	26.9	—
	Proposed system	29.31	49.11

**Table 6:** Examples of Cohen's Kappa metric scores of synthetic Twi and monolingual target sentence and various changes on every model.

Model (*n*_block)	Synthetic Twi sentence	Precision score
Example	Twi monolingual sentence: Mfitiaseɛ no onyankopɔn bɔɔ ɔsoro ne asase	
	Meaning: in the beginning, God created heaven and earth	

Model 1	na baa na baa na baa na baa na baa na baa na baa. (the cane the cane the cane)	0
Model 2	Aseɛ me bɔɔ bɔɔ bɔɔ bɔɔ bɔɔ bɔɔ. (under i created created created created)	0.167
Model 3	Mfitiaseɛ no bɔɔ ne asase. (in the beginning created earth)	0.283
Model 4	Onyankopɔn no ba ɔsoro ma asase. (God come heaven for earth)	0.529
Model 5	Onyankopɔn bɔɔ ɔsoro ne asase. (God created heaven for earth)	0.684
Model 6	Mfitiaseɛ onyankopɔn bɔɔ ɔsoro asase. (in the beginning God created heaven earth)	0.971
Model 7	Mfitiaseɛ no onyankopɔn bɔɔ ɔsoro ne asase (in the beginning, God created heaven and earth)	1

## Data Availability

The parallel corpora are publicly released online for noncommercial use.
